# That was then, this is now: the development of our knowledge and understanding of P2 receptor subtypes

**DOI:** 10.1007/s11302-021-09763-0

**Published:** 2021-02-01

**Authors:** Charles Kennedy

**Affiliations:** Strathclyde Institute of Pharmacy & Biomedical Sciences, University of Strathclyde, John Arbuthnott Building, 161 Cathedral St, Glasgow, G4 0RE Scotland, UK

**Keywords:** P2 receptors, P2X receptors, P2Y receptors, Ligand-gated cation channel, G protein–coupled receptor, Heterodimer

## Abstract

P2 receptors are present in virtually all tissues and cell types in the human body, and they mediate the physiological and pharmacological actions of extracellular purine and pyrimidine nucleotides. They were first characterised and named by Geoff Burnstock in 1978, then subdivided into P_2X_ and P_2Y_ purinoceptors in 1985 on the basis of pharmacological criteria in functional studies on native receptors. Molecular cloning of receptors in the 1990s revealed P2X receptors to comprise seven different subunits that interact to produce functional homo- and heterotrimeric ligand-gated cation channels. A family of eight P2Y G protein–coupled receptors were also cloned, which can form homo- and heterodimers. Deep insight into the molecular mechanisms of agonist and antagonist action has been provided by more recent determination of the tertiary and quaternary structures of several P2X and P2Y receptor subtypes. Agonists and antagonists that are highly selective for individual subtypes are now available and some are in clinical use. This has all come about because of the intelligence, insight and drive of the force of nature that was Geoff Burnstock.

## Introduction

**Fig. 1:**
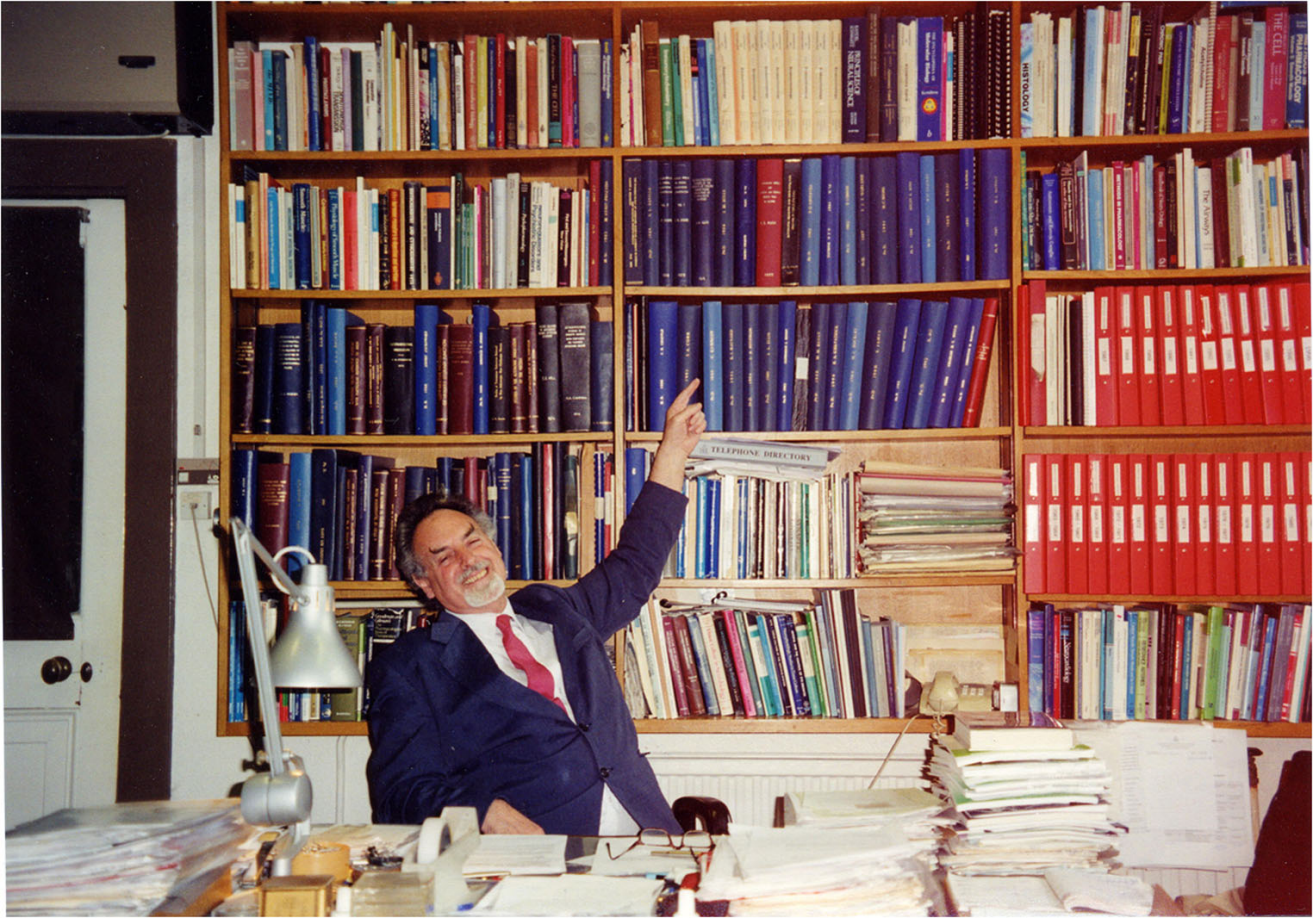
Geoff Burnstock. Geoff Burnstock is seen at his desk in his office at UCL in 1997 pointing at the author’s PhD thesis

I first met Geoff Burnstock on 5 May 1981, when he interviewed me for a PhD position. At the time I was about to complete my undergraduate degree in pharmacology at Aberdeen University. I had come across purinergic neurotransmission in the gastrointestinal tract 3 months previously in a module on non-adrenergic, non-cholinergic neurotransmission. It was love at first sight. I now knew what and where I wanted to study for a PhD. I wrote to Geoff to enquire if he had any positions open and so in May found myself sitting at his desk at UCL, an impressive display of PhD theses behind him (Fig. 1). He was immediately welcoming and enthusiastic and made me feel at ease, and soon this “interview” switched from him asking me questions and me answering, to him telling me, at some length, about what most interested him and where he felt that purinergic research was heading. This was the ideal interview for someone who was quiet and not always forthcoming in interview situations. I got the job.

I spent 4 years at UCL and learned so much from Geoff during that time, not just about science itself but also about what is needed to generate good data and good ideas and how to best promote them to the outside world. Because of him, I read widely, not just purinergic research papers but also wider aspects of neuroscience, pharmacology physiology and biochemistry. Indeed, his breadth of interests and knowledge, along with his energy and drive, explains why he was the most highly cited scientist in the world in pharmacology and toxicology for 12 years, publishing more than 1550 papers that were cited more than 125,000 times and generating an h-index of 156. I also learned that science is a small world and learned about the benefits of collaboration. At any one time, there was somewhere around 18–36 people working with him. As well as numerous PhD students, post-doctoral researchers and research technicians, there were many international visitors. Some came for a week, others for longer. This generated a diverse, ever-changing and lively group. It was a great environment in which to work, and I am forever grateful to Geoff for the opportunity and experience that he gave me.

## That was then—purinoceptors in the early 1980s

### P_1_ and P_2_ purinoceptors

Although adenosine 5′-triphosphate (ATP) and related nucleotides were first isolated [1, 2] and shown to be pharmacologically active [3] in 1929, it was nearly 50 years later before the receptors through which they act were classified on the basis of pharmacological criteria. In 1978, Burnstock proposed that ATP, adenosine 5′-diphosphate (ADP), adenosine 5′-monophosphate (AMP) and adenosine act at P_1_ and P_2_ purinoceptors, as follows [4]. P_1_ purinoceptors were selectively stimulated by adenosine and AMP, leading to changes in intracellular cAMP levels, and were selectively antagonised by methylxanthines, such as theophylline. P_2_ purinoceptors, on the other hand, were selectively stimulated by ATP and ADP, had no effect on intracellular cAMP levels and were unaffected by methylxanthines. This proposal of what was in effect separate adenosine receptors and ATP receptors was generally well-received and widely accepted.

Not long afterwards, it was proposed, on the basis of pharmacological, biochemical and receptor-binding data, that the P_1_ purinoceptor could be subdivided into A_1_ and A_2_ adenosine receptors [5, 6]. A major advantage held by those in the P_1_ purinoceptor field was the early and progressive development of agonists and particularly antagonists that acted selectivity between different adenosine receptor subtypes. This ensured that our knowledge and understanding of the number of P_1_ purinoceptor subtypes and their pharmacological properties were much more advanced than our understanding of P_2_ purinoceptors.

### P_2_ purinoceptor agonists and antagonists

In the early 1980s, the choice of commercially available and useful P_2_ agonists was limited to ATP, ADP and the enzymatically stable analogues, α,β-methyleneATP and β,γ-methyleneATP. 2-MethylthioATP had been synthesised and its effects reported [7, 8], but it was not commercially available. The options for antagonising P_2_ purinoceptors were even more limited. David Westfall’s group had reported that ANAPP_3_ inhibited some of the effects of ATP, including purinergic neurotransmission in the vas deferens [9–11]. ANAPP_3_ was not, however, user-friendly, as it had to be irradiated by a tungsten halogen projector lamp for 20 min, which generated very high local temperatures; its effects were irreversible and it was not available commercially. Apamin, a peptide neurotoxin found in bee venom, had recently been shown to block some of the actions of ATP, particularly purinergic effects in the gastrointestinal tract [12], but it was subsequently shown to block small conductance Ca^2+^-dependent K^+^ channels that are activated by ATP in some tissues.

The first major breakthrough regarding inhibition of P_2_ purinoceptors by a commercially available compound was made by Lubo Kasakov, who was undertaking a sabbatical from the Bulgarian Academy of Sciences with Geoff. Lubo had a long-standing interest in the role of ATP and P_2_ purinoceptors in the atropine-resistant, neurogenic contractions of the urinary bladder. He was characterising the contractile actions of the agonist α,β-methyleneATP, which was much more potent than ATP, in strips of guinea pig urinary bladder, and saw that the contractions were transient. Furthermore, reproducible contractions could only be evoked if an extended interval was left after the washout of α,β-methyleneATP before it was reapplied. Consequently, Lubo hypothesised that this desensitising action of α,β-methyleneATP could be used to study the contribution of P_2_ purinoceptors to parasympathetic neurotransmission in the urinary bladder. His subsequent experiments demonstrated clearly that repeated administration of α,β-methyleneATP depressed the atropine-resistant component of neurogenic contractions [13]. Contractions evoked by exogenous ATP, but not acetylcholine or histamine, were also inhibited by α,β-methyleneATP pretreatment, showing that the inhibition was selective for P_2_ purinoceptors. This was the first clear demonstration that ATP and acetylcholine are cotransmitters from parasympathetic nerves.

Although it was expensive, α,β-methyleneATP was potent and easy to use and so was the first useful, selective P_2_-inhibitor. Consequently, it was soon employed to study the actions of ATP in other tissues. For example, Lorna Meldrum, who worked on an experimental setup next to Lubo Kasakov, shortly afterwards used it to show that ATP mediated a substantial part of the initial phasic contraction of sympathetic nerve–mediated contractions of the guinea pig vas deferens [14]. This agreed with the earlier data obtained using ANAPP_3_ and confirmed that ATP and noradrenaline are cotransmitters from sympathetic nerves. α,β-MethyleneATP-induced desensitisation of P_2_ purinoceptors was also used to demonstrate that ATP is a cotransmitter in vascular sympathetic nerves [15, 16]. Thereafter, α,β-methyleneATP was employed in many studies on the actions of ATP, particularly as a cotransmitter [ see 17, 18].

### Experiments leading to the recognition of P2 receptor subtypes

As was often the case with Geoff, the subject of my PhD was not rigidly defined. Instead, he made several suggestions for potential experiments and told me to read the literature and identify gaps in our knowledge and understanding. I was expected to then find and develop *something interesting* about P_1_ and P_2_ purinoceptors. Initially, I performed some experiments using the guinea pig taenia coli, the tissue that had contributed so much to me being at UCL, but they came to nothing and I moved on to portal vein, which, at that time, in the rabbit was the best example of purinergic inhibitory neurotransmission outside of the gastrointestinal tract [19, 20]. These experiments produced two papers on the modulatory actions of purines on sympathetic neurotransmission [21, 22], but they did not meet the challenge of being *interesting*.

As I entered the final year of my PhD, I realised that although numerous studies had been published relating to the pharmacological properties of P_2_ purinoceptors present in the vas deferens and urinary bladder smooth muscle from a variety of species, the same was not true for the vascular smooth muscle. Also, endothelium-dependent vasodilation had recently been discovered [23], and there was only one report of endothelium-dependent effects of ATP [24]. Thus, a gap in our knowledge and understanding had been identified, and I decided to characterise the pharmacological profile of the P_2_ purinoceptors that mediate contraction and relaxation of vascular smooth muscle.

**Fig. 2:**
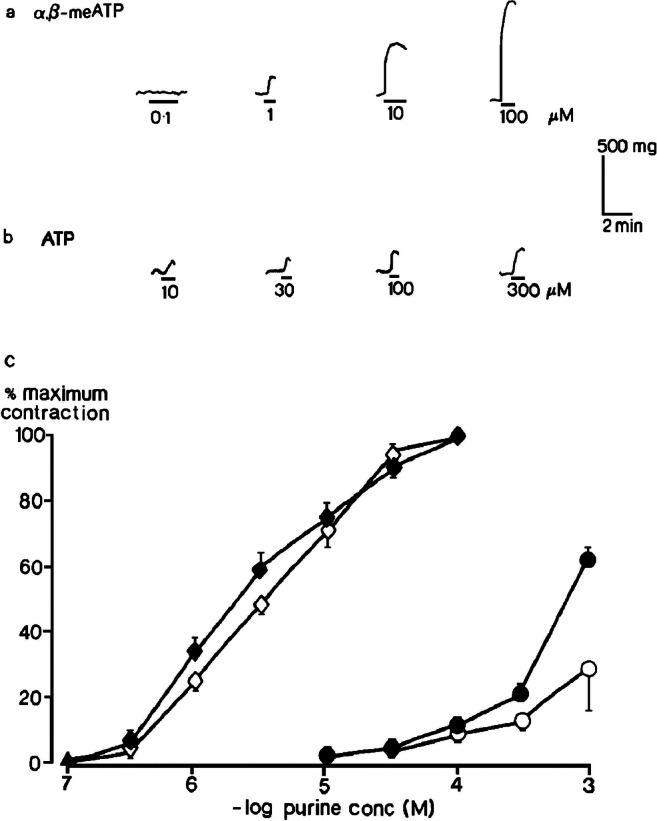
Contractions of rat isolated femoral artery. Contractions evoked by (**a**) α,β-methyleneATP (10^−7^–10^−4^ M) and (**b**) ATP (10^−5^−3 × 10^−4^ M) at resting tone when endothelium was intact are shown; (**c**) log concentration-response curves for contractions evoked by α,β-methyleneATP (10^−7^–10^−4^ M) (◊,♦) and ATP (10^−5^–10^−3^ M) (○,●) at resting tone when endothelium was intact (open symbols) or removed (closed symbols) (*n* = 6) are shown. Vertical bars show sem. Reproduced from [25] (Kennedy et al., 1985, with permission from Elsevier)

**Fig. 3:**
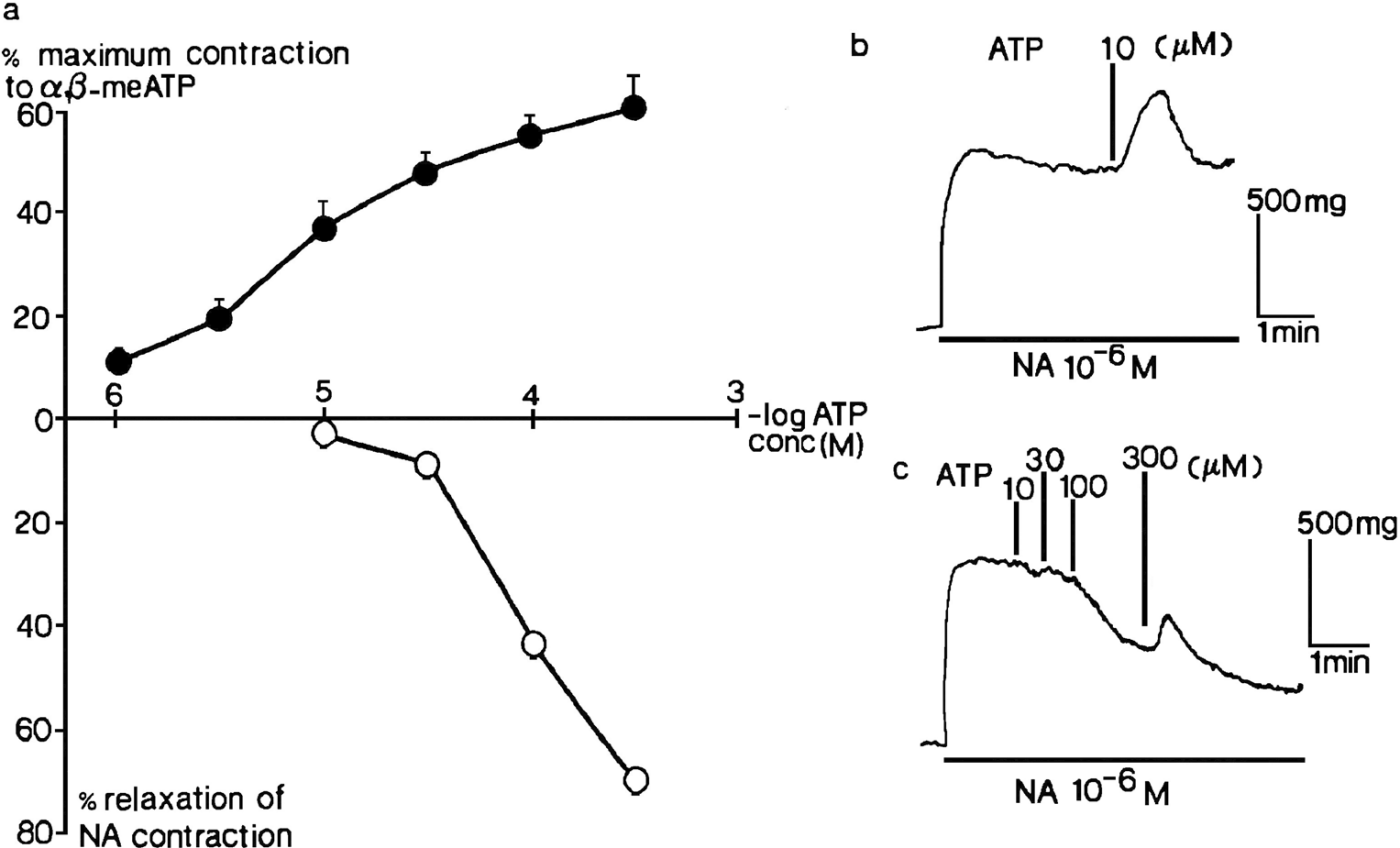
Two types of response to ATP in rat isolated femoral artery. **a** The effects of ATP (10^−6^–3 × 10^−4^ M) in tissues precontracted by 10^−6^ M noradrenaline (NA) when endothelium was intact (o) or removed (●) (*n* = 4) are shown. Vertical bars represent sem. **b** Endothelium removed, contraction to 10^−5^ M ATP. **c** Endothelium intact, relaxations to ATP (10^−3^–3 × 10^−4^ M). Reproduced from [25] (Kennedy et al., 1985, with permission from Elsevier)

**Fig. 4:**
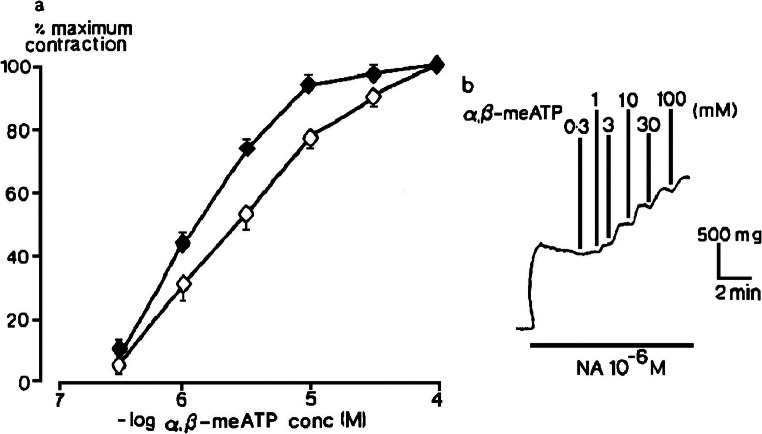
One type of response to α,β-methyleneATP in rat isolated femoral artery. **a** The effect of α,β-methyleneATP (3 × 10^−7^–10^−4^ M) in tissues precontracted by 10^−6^ M noradrenaline (NA) when endothelium was intact (o) or removed (●) (*n* = 6) are shown. Vertical bars show sem. **b** Contractions evoked by α,β-methyleneATP when the endothelium was intact are shown. Reproduced from [25] (Kennedy et al., 1985, with permission from Elsevier)

Experiments in the rat isolated femoral artery found that at the resting tone, α,β-methyleneATP evoked concentration-dependent contractions, with an EC_50_ of ~4 μM (Fig. 2) [25]. ATP acted likewise, but was much less potent, and a clear maximum to its concentration-response curve was not reached. Concomitant experiments in the rabbit isolated ear artery found a similar large difference in the relative potency of α,β-methyleneATP and ATP at inducing vasoconstriction [26]. To examine vasodilatory actions, muscle tone was first raised by noradrenaline. Now, ATP elicited relaxation at low concentrations that had had no effect at resting tone, and transient contraction followed by a maintained relaxation at higher concentrations (Fig. 3). The relaxations were abolished by physically removing the endothelial cells, and now only contractions were seen. α,β-MethyleneATP, in contrast, never evoked vasodilation, either in the absence or in the presence of the endothelial layer in the femoral artery (Fig. 4) or ear artery [26]. As a pharmacologist, it was clear to me that the pharmacological properties of the smooth muscle P_2_ purinoceptors were different from those of P_2_ purinoceptors on the endothelium.

**Fig. 5:**
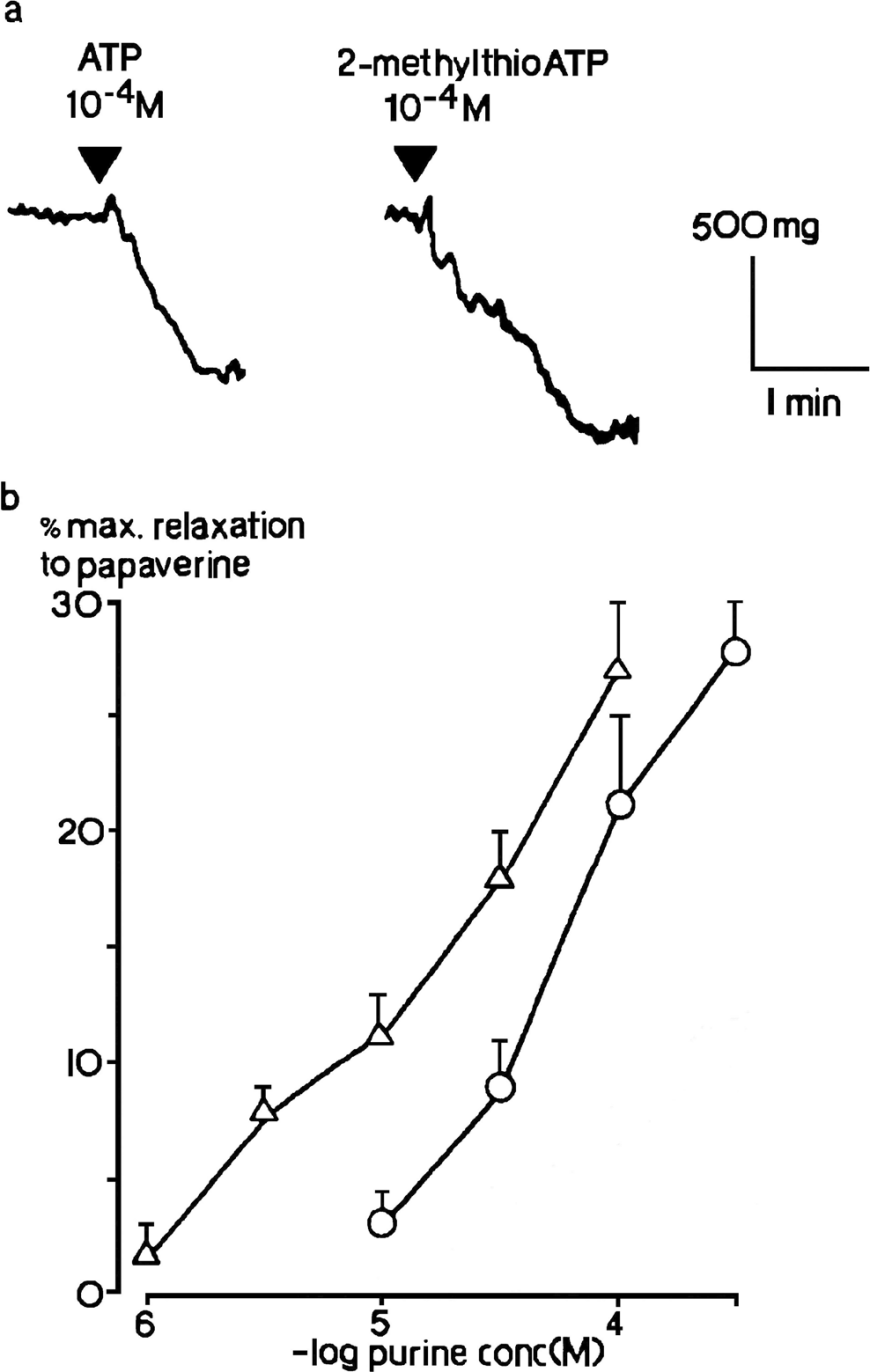
Relaxations of rat isolated portal vein longitudinal muscle. **a** Relaxations of precontracted tissues induced by 2-methyithioATP (10^−4^ M) and ATP (10^−4^ M) in the same preparation are shown. **b** The mean peak amplitude of relaxations evoked by 2-methylthioATP (10^−6^–10^−4^ M) (Δ) and ATP (10^−5^-3 × 10^−4^ M) (O) (*n* = 6) are shown. Vertical bars show sem. Adapted from [26] (Kennedy and Burnstock, 1985, with permission from Elsevier)

Further experiments were performed using the rabbit portal vein longitudinal muscle layer, where ATP was proposed to be an inhibitory neurotransmitter. In tissues precontracted by ergotamine, ATP elicited relaxation (Fig. 5), but α,β-methyleneATP evoked contraction [27]. I also had a great stroke of luck at this point when, at the back of a packed freezer, I found a single vial of 2-methylthioATP stock solution, which had been synthesised by Noel Cusack, a chemist at King’s College London, for an earlier study [28]. 2-MethylthioATP also evoked relaxation and was more potent than ATP (Fig. 5). Talking with Noel a few years later, we came to the conclusion that this may have been the only sample of 2-methylthioATP available anywhere in the world at that time.

The data from these studies indicated two very different P_2_ purinoceptor profiles. At one site, α,β-methyleneATP evoked contraction and was much more potent than ATP, whilst at the other, 2-methylthioATP was more potent than ATP at evoking relaxation, and α,β-methyleneATP was inactive. I thought that I had discovered *something interesting*, and when I showed the data to Geoff, it was clear from his excitement that I had.

### P_2X_ and P_2Y_ purinoceptors

At this point we re-examined the published literature with these two P_2_ purinoceptor profiles in mind and identified numerous reports, which contained individual pieces of data that were consistent with our hypothesis that P_2_ purinoceptors did not comprise a single homogenous group. These included differences in the relative potency of agonists in visceral and vascular smooth muscle and cardiac muscle preparations, differences in the degree of stereo-selectivity displayed by stereo-isomers of P_2_ agonists and differences in the ability of ANAPP_3_ or desensitisation of P_2_ purinoceptors to inhibit agonist-induced responses. This led us to ask “Is there a basis for distinguishing two types of P_2_-purinoceptor?” [29].

The strongest evidence was that α,β-methyleneATP and β,γ-methyleneATP were more potent than ATP, which was equipotent with 2-methylthioATP, when contraction of smooth muscle tissues, such as the guinea pig isolated vas deferens and urinary bladder and arteries, was measured. On the other hand, 2-methylthioATP was more potent than ATP, which in turn was more potent than α,β-methyleneATP when the relaxation of rabbit isolated portal vein, pig isolated aorta and guinea pig isolated taenia-coli was measured. In addition, prolonged administration of α,β-methyleneATP or pretreatment with ANAPP_3_ inhibited contractions of the vas deferens and urinary bladder, but had no effect on relaxations of the portal vein or taenia-coli. On the basis of our own and the published data, we proposed the existence of P_2X_ and P_2Y_ purinoceptors.

These names were chosen because we thought that they would be easy to remember. Greek letters have been used to label subtypes of other receptors, for example μ, δ, and κ in the opioid field and α and β in the adrenoceptor field, but we chose not to follow suit. Of the remaining Greek symbols that are not in common use, how many of us can remember how to pronounce ξ, ζ or ν?

In hindsight, we probably should have used the terms P_2A_ and P_2B_ purinoceptor, which would have avoided the subsequent “random walk through the alphabet” that occurred when further subtypes were proposed, but the P_2X_ and P_2Y_ nomenclature has survived to this day.

Over the next few years, the concept of P_2_ purinoceptor subtypes gained support as their pharmacological properties in other tissues were characterised. Evidence was also published that the agonists, β,γ-methylene-L-ATP and ADP-β-F, displayed selectivity for P_2X_ and P_2Y_ purinoceptors respectively. It would be some time before subtype-selective antagonists also became available, but the introduction of suramin as the first, easy to use, non-selective P_2_ purinoceptor antagonist was a major development for purinergic research in general. In addition, it became apparent from a variety of approaches that P_2X_ purinoceptors are ligand-gated cation channels (LGIC), and P_2Y_ purinoceptors are G protein–coupled receptors (GPCR). That the pharmacological division was mirrored by a division in molecular structure is not surprising, as it was well-understood by that time that P_2X_ purinoceptors mediate the fast neurotransmitter actions of ATP and so were likely to be ion channels, whereas P_2Y_ purinoceptors produced their effects over a slower time-course, indicating activation of second messenger signalling pathways.

### More P_2_ purinoceptor subtypes

Shortly afterwards, John Gordon proposed two further subtypes, P_2T_ purinoceptors, which are present in platelets and mediate aggregation, and P_2Z_ purinoceptors, expressed in mast cells, mediating degranulation [30]. Uracil nucleotides, such as uridine 5′-triphosphate (UTP), were also known to be pharmacologically active, and a separate pyrimidoceptor was proposed [31]. Subsequently, the P_2U_ purinoceptor, which was activated by both ATP and UTP, was identified [32]. Finally, the P_2D_ purinoceptor was named as a subtype through which adenine dinucleotides elicit some of their pharmacological effects [33, 34].

## This is now—P2 receptors in the 2020s

### The cloning of P2 receptors

Starting in 1993, seven ATP-sensitive LGIC subunits and eight adenine and/or uracil nucleotide-sensitive GPCR were cloned [35, 36]. Subsequently, in recognition that pyrimidines, as well as purines, were pharmacologically active and that there was a degree of overlap in their sites of action, it was agreed that the term *P*_*2*_ *purinoceptor* be dropped and instead that all of the nucleotide-sensitive subtypes be referred to as *P2 receptors* (P2R). It was also agreed that all LGIC, including the P_2Z_ purinoceptor, be referred to as P2X receptors (P2XR) and all GPCR, including the P_2U_ purinoceptor, be referred to as P2Y receptors (P2YR). Thus, cloning greatly simplified the P2R nomenclature.

The *Ensembl Gene* database shows that to date, 151–350 species orthologues of each P2XR subunit and 191–295 of each P2YR have been cloned. They are all present in placental mammals, as well as in birds, reptiles, fish and primitive organisms, such as amoeba and algae, but interestingly, not *Caenorhabditis elegans*, *Drosophila melanogaster* or *Saccharomyces cerevisiae*. The P2Y_11_R is notable for also not being present in rats and mice [37].

Each P2XR subunit comprises a single polypeptide chain that forms two helical transmembrane spanning regions (TMR), with a large extracellular loop and intracellular N- and C-termini. The human P2X1R to P2X6R are 379–472 amino acids long, whilst the P2X7R is 595 amino acids long and has a much larger intracellular C-terminus. All, apart from the P2X6R subunit, form homomeric, non-selective cationic channels, with a relatively high permeability to Ca^2+^. As well as forming homomers, subunits can also interact with each other to form heteromultimers. At present, seven functional heteromultimers have been identified (P2X1/2R, P2X1/4R, P2X1/5R, P2X2/3R, P2X2/6R, P2X4/6R and possibly P2X4/7R), which have different pharmacological and/or biophysical properties from the individual homomultimers [38].

P2YR belong to the δ group of class A, rhodopsin-like GPCR and are also single polypeptide chains of 328–377 amino acids that form seven TMR, with an extracellular N-terminus and intracellular C-terminus. As GPCR, they couple to and activate heterotrimeric G proteins. Principally, the P2Y_1_R, P2Y_2_R, P2Y_4_R, P2Y_6_R and P2Y_11_R subtypes couple to Gα_q/11_, whilst the P2Y_12_R, P2Y_13_R and P2Y_14_R couple to Gα_i/o_. The P2Y_11_R also couples to Gα_s_.

Several recent comprehensive reviews describe the properties of the recombinant P2XR [39, 40] and P2YR [41, 42] in detail. I will, therefore, focus here on recent developments in our knowledge and understanding of their structure, pharmacological properties and potential for therapeutic application.

### P2XR structure

#### Tertiary and quaternary structures

**Fig. 6:**
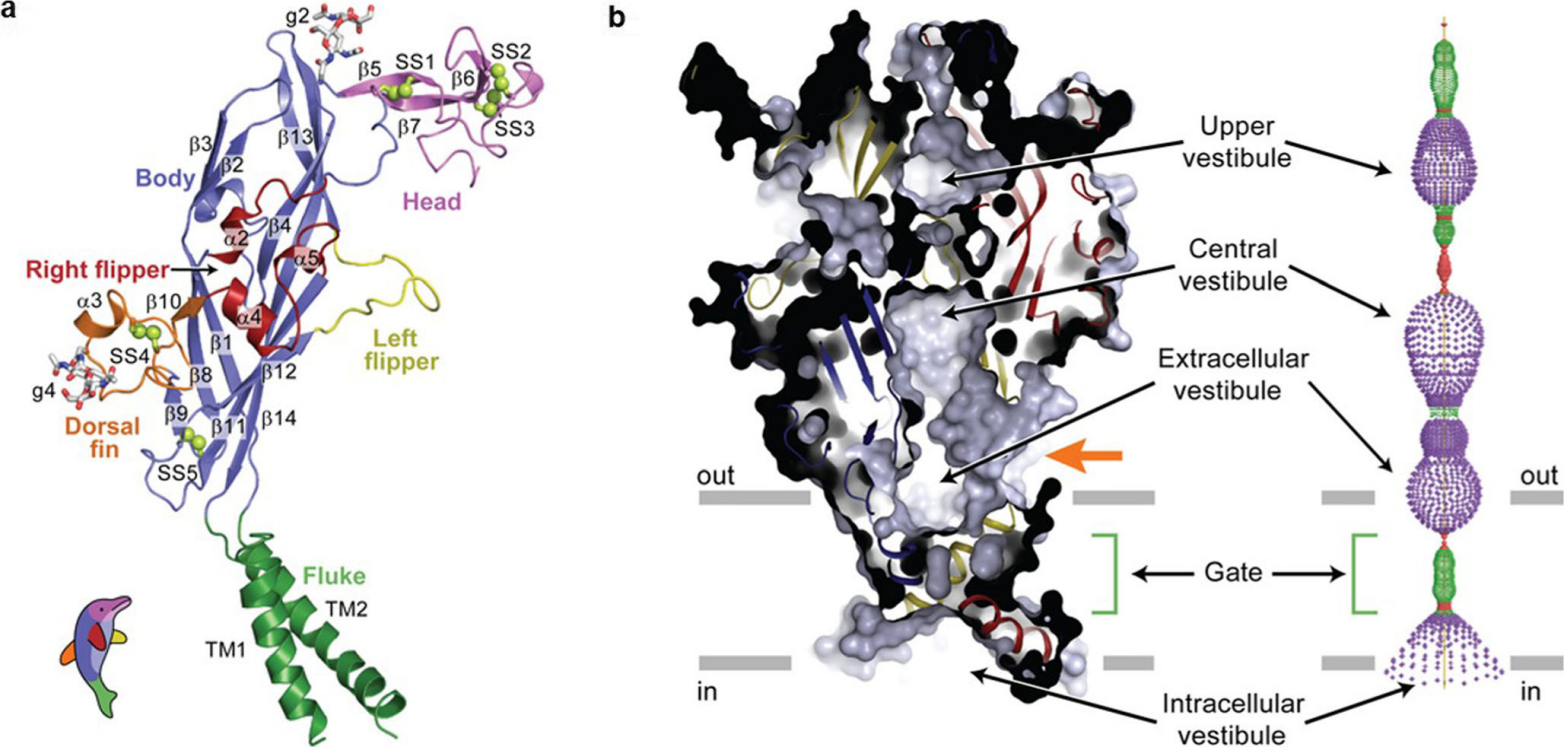
Subunit fold and closed, resting conformation of zebrafish P2X4.1 receptor. **a** The ΔzfP2X4 subunit has a dolphin-like shape. Alpha helices (TM1–2 and α2–5), beta strands (β1–14), disulphide bonds (SS1–5) and attached glycans (g2 and 4) are indicated. **b** Left-hand panel: a sagittal section reveals a closed conformation of the pore and shows that the gate is located about halfway across the membrane bilayer. Three vestibules (upper, central and extracellular vestibules) are located on the molecular 3-fold axis, with the extracellular vestibule connected to the bulk solution through a fenestration (orange arrow). Right-hand panel: pore lining surface calculated by the Hole49 program. Each colour represents a different radius range measured from the receptor centre (red: <1.15 Å, green: 1.15–2.3 Å, and purple: >2.3 Å). (Reproduced from [42] Kawate et al., 2009, with permission from the Nature Publishing Group)

The first three-dimensional structure, a truncated mutant of the zebrafish p2X4.1R, was revealed in 2009 by X-ray crystallography at a resolution of 3.5 Å (Fig. 6) [43, 44]. Now, 27 high-resolution zebrafish [45, 46], human P2X3R [47–49], rat P2X7R [50], giant panda P2X7R [51], chicken P2X7R [52] and the Gulf Coast tick *Amblyomma maculatum* P2XR [53] structures have been reported. They confirm the conclusions of earlier indirect structural studies that three subunits interact to form a functional receptor. Each subunit adopts a conformation that resembles the shape of a leaping dolphin (Fig. 6a). The tail represents the TMR, the upper body the bulk of the extracellular loop and the head the most distal part of the extracellular loop.

The subunits wrap round each other to produce a structure that resembles a chalice (Fig. 6b). The large extracellular domain protrudes above the plasma membrane plane by ~70 Å and contains numerous β-strands and conserved, interacting cysteine residues, which give the structure rigidity. The TMR are α-helices that extend across the plasma membrane at an angle of nearly 45° relative to its plane. The intracellular N- and C-termini of the zebrafish p2X4.1R were truncated to improve crystallisation, but studies on a human P2X3R construct in which there is less truncation show that they are flexible and disordered in the apo, closed state, but form a “cytoplasmic cap” of highly intertwined β-sheets just under the plasma membrane that stabilises the open state [47, 49]. In contrast, the cytoplasmic cap of the full-length rat P2X7R can be seen in both the apo and open states [50]. This may be a basic difference between desensitising and non-desensitising P2XR.

The C-terminus of the rat P2X7R also contains an 18-amino acid long, cysteine-rich region, the C-cys anchor, at the cytoplasmic end of TMR2, which interacts with the N-terminus of an adjacent subunit and links TMR2 to the cytoplasmic cap. The C-cys anchor contains at least four cysteine residues and one serine residue that are palmitoylated and the aliphatic chains extend into the plasma membrane, anchoring the receptor to the membrane. This might keep the cytoplasmic cap in place and so limit P2X7R desensitisation. Consistent with this, the receptor desensitised rapidly and fully when the C-cys anchor was deleted or the cysteine residues removed by mutation. A further unique feature of the rat P2X7R is the long C-terminal (~200 residues), term “cytoplasmic ballast”. Each receptor has three globular, wedge-shaped cytoplasmic ballasts that hang beneath the TMR of an adjacent subunit. Intriguingly, each cytoplasmic ballast contains a dinuclear Zn^2+^ complex and a high-affinity guanosine nucleotide binding site, the functions of which are unclear.

Two pathways are apparent along which extracellular cations may diffuse to reach the transmembrane pore. Immediately above the TMR, there is an extracellular vestibule from which three fenestrations, of up to 8 Å in diameter, form a short pathway, as indicated by the orange arrow in Fig. 6b. The second route is much longer and runs the length of the extracellular domain. This pathway passes through the extracellular vestibule and two further vestibules that are lined with negatively charged residues that may attract Na^+^ and Ca^2+^ and so facilitate ion movement. An inner vestibule shaped like an inverted cone lies on the cytoplasmic side of the channel gate. Beneath it sits the cytoplasmic cap, which has lateral, phospholipid-lined cytoplasmic fenestrations that are routes for ions to enter and exit the pore [47, 50].

#### Agonist binding site

Three orthosteric ATP-binding pockets are present in the grooves formed by the sites of interaction between the three subunits and located ~40 Å above the plasma membrane plane [45]. When bound, the adenine base of ATP interacts with conserved charged lysine and polar threonine residues and hydrophobic leucine and isoleucine residues deep in the binding pocket. The ribose ring binds to a leucine, and the phosphates bind to several highly conserved, positively charged lysine and asparagine residues and a polar arginine residue. These interactions cause ATP to adopt a U-shaped structure, with its β- and γ-phosphates folded towards the adenine base. 2-MethylthioATP [47] and CTP [46] also bind within the orthosteric pocket and in a similar manner to ATP, although CTP has fewer interactions, which may account for its much lower potency at P2XR. It is notable that the entrance to the binding pocket is much narrower in P2X7R (<11 Å orifice) [50] compared to P2X3R (17 Å orifice) [47]. This and any protein flexibility that opens and closes the entrance would decrease the time ATP spends in the binding pocket, so decreasing its affinity, which could explain the three orders of magnitude lower potency of ATP at P2X7R compared to other P2X subtypes.

### P2XR receptor gating

The initial data on the zebrafish p2X4.1R were obtained in the absence of ATP and represent the closed state of the channel. TMR2 was confirmed as forming the channel, which is blocked by a series of mainly hydrophobic residues over two turns of the TMR2 α-helix and about 8 Å in length. Ala344 is at the centre of this gate region and is the point at which the TMR2 are closest. This configuration constricts the pore, giving it the appearance of an hourglass (Fig. 6b). There are fewer subunit interactions in and around the TMR, which enables the TMR to move relative to each other when ATP binds and so opens the pore.

High-resolution structures of agonist-bound open channel and desensitised, closed channel configurations reported since then [45–47, 49, 50] indicate that ATP causes the binding pocket to tighten. This leads to outward expansion of the six TMR and change of the pitch of each TMR2 from an α-helix to a 3_10_-helix, which in turn causes channel opening. As discussed above, the cytoplasmic cap stabilises non-desensitising P2XR, but disassembles in desensitising P2XR, producing a closed, desensitised state.

#### Antagonist binding sites

The competitive antagonists, TNP-ATP and A-317491, bind within the P2X3R orthosteric site, but at a deeper position than ATP, and they adopt a Y-shape [47]. TNP-ATP also binds within the P2X7R orthosteric site, but in an extended conformation [52]. The structural constraints on antagonist binding are lower than on agonist binding, which is not surprising given that antagonists only have to block the access of an agonist to the binding site, whereas agonists must bind in a precise, well-defined manner that is sufficient to induce conformational change and so opening of the pore.

Allosteric binding sites have also been identified. Five structurally unrelated P2X7R negative allosteric modulators (NAMs), including A740003, bound in a groove formed between neighbouring subunits, above the orthosteric binding pocket [51]. In contrast, the P2X3R NAM, AF-219, bound to a pocket formed by the lower body and dorsal fin of one subunit and the lower body and left flipper of an adjacent subunit and which is closer than the orthosteric site to the plasma membrane [48]. It is likely that the NAMs limit or prevent the mechanical rearrangements that underlie receptor activation and pore opening.

### Subtype-selective agonists

At present, no highly subtype-selective agonists are available, which is perhaps unsurprising in view of the highly conserved nature of the ATP-binding pocket. α,β-MethyleneATP was initially reported to only stimulate homomeric P2X1R and P2X3R, as well as at heteromeric P2X4/6 R and P2X1/5R, but was then shown to also have agonist activity at P2X4R, P2X5R, P2X6R and P2Y_11_R [54]. Similarly, β,γ-methylene-l-ATP is a potent and stable partial agonist at P2X1R, P2X3R and P2X5R. Its action is stereo-selective at P2X3R (d > > l), but not P2X1R (d = l). It has little or no known effects at other P2XR and P2YR. BzATP is sometimes described as a selective agonist for P2X7R, but in fact, its affinity is the highest for the P2X1 subtype [55]. At one point, it was hoped that diadenosine polyphosphates (APnA) might display P2X subtype selectivity, but on the whole, this has not proved to be the case, as AP_6_A, AP_5_A and AP_4_A are agonists at most P2X subtypes studied [see 56, 57].

The best approach for producing selective stimulation of P2X subtypes may be through the development of positive allosteric modulators (PAMs). The best characterised example is ivermectin, which potentiates ATP-induced ionic currents through P2X4R [58]. Interestingly, it has the same effect in humans, but not rodent P2X7R [59]. Other selective PAMs include MRS2219 at P2X1R and GW791343 and clemastine and polymyxin B at P2X7R [39]. PSB-10129 is a PAM of P2X2R, cibacron blue potentiates agonism of P2X3R and P2X4R and ginsenosides; the main constituents of ginseng are PAMs at P2X4R, but their selectivity is not yet known [40].

#### Subtype-selective antagonists and therapeutic indications

##### P2X3R antagonists

Greater progress has been made in the development of P2XR subtype-selective antagonists, particularly for P2X3R and P2X7R, and many of them are commercially available [39, 40, 60]. A-317491 was the first selective P2X3R antagonist and was followed by others with improved potency, solubility and pharmacokinetic properties, such as AF-353, AF-906, RO-3, RO-85 and BLU-5937. At present, the most promising clinical target appears to be chronic cough. In a recent phase 2b clinical trial in patients with refractory chronic cough or unexplained cough, Gefapixant (AF-219, MK-7264), named after Geoff Burnstock, reduced the awake cough frequency in a dose-dependent manner [61]. Dysgeusia, reduced taste sensitivity, was seen in some patients and also appeared to be dose-dependent. It is probably due to the inhibition of P2X2/3R in taste buds. DT-0111 is another P2X2/3R antagonist that is water-soluble and so suitable for administration by inhalation and under consideration for clinical use [62]. BLU-5937 is a selective P2X3R antagonist that does not appear to affect taste [63] and is also in phase 2 trials for treatment of refractory chronic cough [64].

Neuropathic pain is also of interest, as a substantial body of evidence indicates roles for P2X3R and P2X2/3R in the primary sensory neurons in this condition, and selective antagonists are effective in animal models [65]. Interestingly, P2X4R in microglia in the spinal cord dorsal horn are also implicated in neuropathic pain, but only in males [66]. A recently developed selective P2X4R antagonist, NC-2600, produced no serious side effects in a phase 1 trial [65].

##### P2X7R antagonists

Numerous potent and selective P2X7R antagonists are now available, including A804598, A839977, A740003, A438079, CE-224535, EVT-401, GSK314181A, JNJ-47965567 and JNJ-54175446 [39, 40, 67]. The subtype selectivity of competitive, orthosteric antagonists can be an issue, and most selective P2X7R antagonists are NAMs, for example AZ11645373, AZ10606120, AZD9056, CE-224,535, GSK1482160 and GW791343. Because of their important role in inflammation [67, 68], P2X7R have therapeutic potential for treating chronic systemic inflammatory diseases, such as rheumatoid arthritis, allergies, asthma, COPD and autoimmune diseases [69] and CNS disorders that have a neuroinflammatory component, e.g. Alzheimer’s disease, major depression and bipolar disorders [40, 68]. This is reflected by the increase in both the number of patents filed and clinical trials registered for P2X7R-targeted treatment [67]. No clear improvement in rheumatoid arthritis patients has been reported so far, though many clinical data are yet to be published. An alternative approach to using receptor antagonists is to instead develop biologics directed against the receptor, and a very potent anti-P2X7R bivalent nanobody-Fc was effective in mouse models of experimental glomerulonephritis and allergic contact dermatitis [70].

There is also great interest in the possibility of targeting P2X7R in cancer, as they are expressed in many types of cancerous cells, and P2X7R antagonists inhibit the growth and metastasis of these cells in preclinical studies [67, 71]. As yet, however, no large-scale clinical trials have tested this hypothesis, but the results of a small phase 1 trial in patients with basal cell carcinoma are promising [72]. They showed that topical application of an ointment containing an antibody, BIL010t, directed against nfP2X7R, a variant of the P2X7R in which the normally hidden E200 epitope is exposed, was well-tolerated and the lesion area was reduced in 65% of patients. An anti-P2X7R vaccine (BIL06v), directed against the same epitope, is also being assessed for safety and immunogenicity in patients with advanced solid tumours [67].

#### P2YR structure

##### Tertiary structures

Determination of the crystal structures of the human P2Y_1_R [73] and P2Y_12_R [74, 75] in ligand-bound states at resolutions of 2.2–3.1 Å provided detailed insight into their tertiary structures and how agonists and antagonists interact with them to produce their effects. They confirm that both subtypes have the canonical seven TMR of GPCR, linked by three extracellular loops (ECL) and three intracellular loops, but they are, nonetheless, structurally distinct. All ECLs and two disulphide bonds, connecting the N-terminus to helix VII and helix III to ECL2, were clear in the P2Y_1_R. This was only the case, however, for the agonist-bound P2Y_12_R, as only ECL3 and the N-terminus-helix VII disulphide bond could be resolved in the antagonist-bound structure. Thus, the ECLs of the P2Y_12_R are likely to be more labile.

Another notable difference is that whereas, in common with most class A GPCR, TMR5 of the P2Y_1_R, contains a proline residue that introduces a bend into the helix, the P2Y_12_R has asparagine in the equivalent position, producing a straight, elongated conformation, which shifts the extracellular end of TMR5 towards helix IV by more than 6 Å. Also, the P2Y_12_R, but not the P2Y_1_R contains a C-terminal helix VIII, which lies parallel to the plasma membrane.

##### Agonist binding site

Thus far, only the P2Y_12_R has been solved in an agonist-bound state [75]. The orthosteric binding site is within the TMR bundle, just beneath the plane of the plasma membrane and bordered by the N-terminus, ECL2, TMR6 and TMR7. The adenine ring of the full agonist, 2-methylthioADP, penetrates furthest into the binding pocket, interacting with Tyr105 in TMR3, whilst the 2-thioether moiety inserts into a hydrophobic pocket formed by TMR3 and TMR4. Above this, the negatively charged phosphates interact with positively charged residues and hydrogen-bonding groups within the N-terminus, ECL2, TMR3, TMR6 and TMR7, rearranging them to form a “lid” over the binding pocket that completely encloses 2-methylthioADP within the binding pocket. As such, this orthosteric binding pocket has a very distinct shape and location compared to those of other GPCR.

#### Antagonist binding sites

##### P2Y_12_R

The non-nucleotide P2Y_12_ antagonist and anti-thrombotic agent, AZD1283, binds within an elongated pocket that stretches by more than 17A° between helices IV and VII and which partially overlaps the binding site of 2-methylthioADP [74]. AZD1283 interacts with some of the same amino acid residues as 2-methylthioADP, for example Tyr105, Phe106 and Lys155, but also has distinct polar and hydrophobic interactions with side chains from helices III–VII. A particularly noticeable difference is that the bulk of AZD1283 prevents the inward movement of helices VI and VII, so preventing closure of the “lid”.

Interestingly, a sub-pocket exists within the ligand-binding cavity of P2Y_12_R that is unoccupied in the solved structures and which includes Cys97 in ECL2. Molecular docking studies suggest that this sub-pocket could interact with the active metabolites of P2Y_12_R antagonists, such as clopidogrel [74]. This is consistent with Cys97 being the site of covalent binding to their thiol moieties [76, 77].

##### P2Y_1_R

**Fig. 7:**
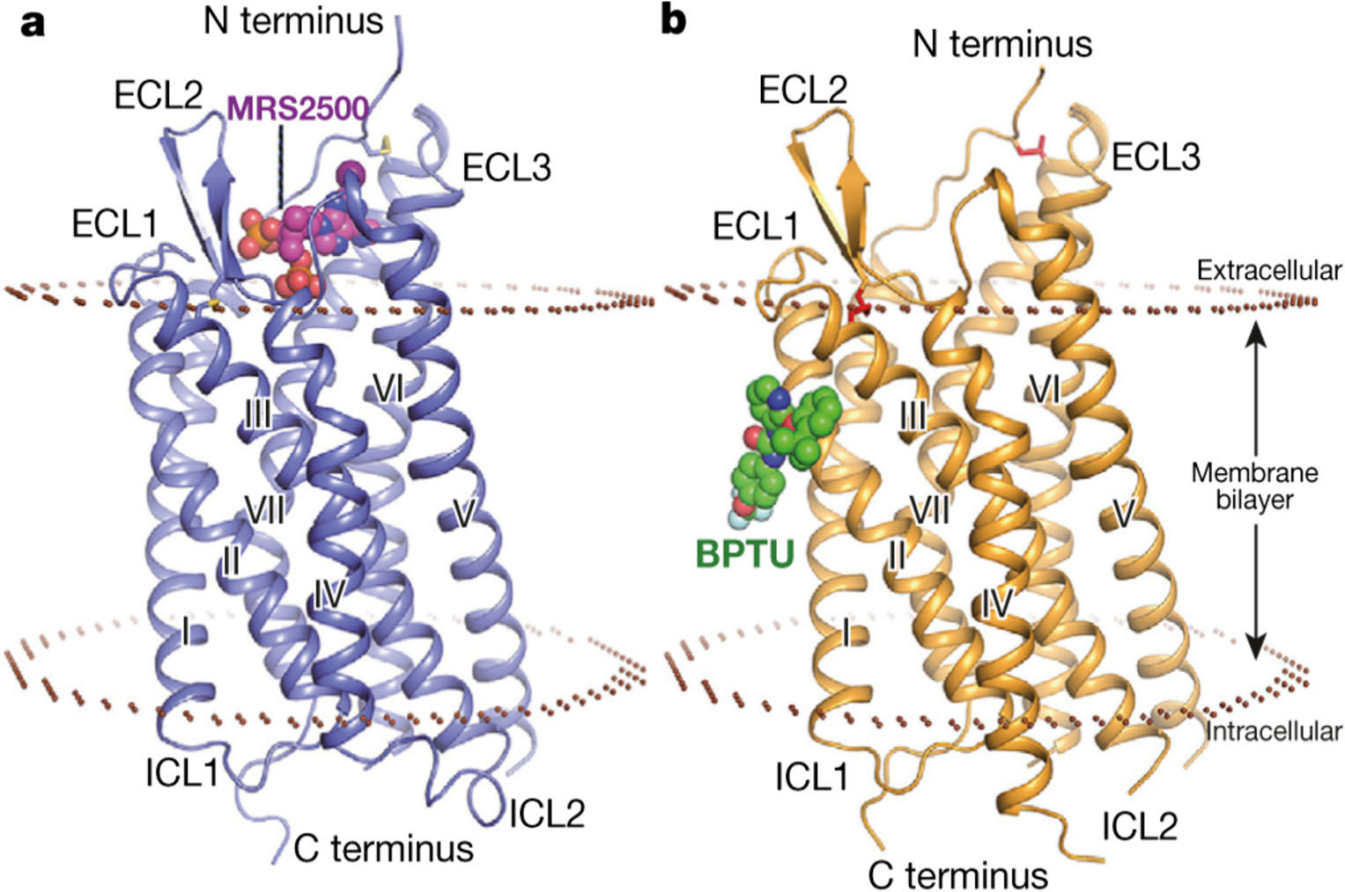
The P2Y_1_R in complex with MRS2500 and BPTU. Side views of the P2Y_1_R in complex with (**a**) MRS2500 and (**b**) BPTU are shown. The receptor is shown as blue (**a**) and orange (**b**) cartoon representation. MRS2500 and BPTU are shown in sphere representation with magenta and green carbons, respectively. The membrane boundaries (brown) are adapted from the OPM database^31^ with P2Y_12_R (PDB ID: 4NTJ) as a model (reproduced from [72] Zhang et al., 2015, with permission from the Nature Publishing Group)

The potent and selective competitive P2Y_1_R antagonist, MRS2500, a bisphosphonate derivative of ADP, binds in a pocket within the TMR bundle and just above the plane of the plasma membrane, which is defined by N-terminus, ECL2, TMR6 and TMR7 (Fig. 7a) [73]. The adenine ring of MRS2500 forms hydrophobic interactions with amino acids within a sub-pocket formed by the N-terminus, TMR6 and TMR7. The 2-iodo group, which is critical for high binding affinity of MRS2500, forms a hydrogen bond with Cys42 in the N-terminus. The two phosphate groups, which are also very important for the activity of MRS2500, form hydrogen bonds and salt bridge interactions with numerous polar residues in the N-terminus, ECL2, TMR2 and TMR7. Thus, although ADP is the endogenous agonist for both subtypes, the orthosteric ligand-binding pocket and mode of ligand interaction of the P2Y_1_R are very different from those of the P2Y_12_R, with only a small degree of overlap of the phosphate binding regions.

A unique binding site for the non-nucleotide NAM, BPTU, was also identified on the outer surface of the P2Y_1_R at its interface with lipids of the plasma membrane (Fig. 7b) [73]. Aromatic and hydrophobic residues in helices I, II and III form a shallow binding pocket and interact with BPTU in a hydrophobic manner. In view of its location, it is likely that BPTU reaches the binding pocket via the lipid bilayer, which is consistent with the high lipophilicity of BPTU. Finally, it is interesting to note that although MRS2500 and BPTU have very different binding sites, they both stabilise the P2Y_1_R in similar inactive conformations.

### P2YR oligomers

#### P2YR homodimers

Until quite recently, GPCR were thought to exist as monomeric entities that couple to G proteins on a 1:1 stoichiometric basis, but it is now clear that they can interact to form dimeric or higher-ordered oligomeric complexes that may couple to one or more G proteins [78, 79]. This can result in changes to the subcellular localisation and trafficking of receptors, their pharmacological properties and functional activity. Constitutive formation of recombinant P2Y_1_R, P2Y_4_R, P2Y_6_R, P2Y_12_R, P2Y_13_R and P2Y_14_R homodimers was shown by co-immunoprecipitation or FRET [80–83], and both P2Y_1_R and P2Y_12_R crystallised as homodimers [73]. The P2Y_12_R homodimer appears to be its physiologically active form, as a dominant negative P2Y_12_R variant was identified in a family that suffered from severe bleeding [84]. Also, the active metabolite of the anti-thrombotic P2Y_12_R antagonist, clopidogrel, reduced the P2Y_12_R to monomers, and this was proposed to be its mechanism of action [81].

#### P2YR heterodimers

Physical interaction between different recombinant subtypes also occurs, for example P2Y_4_R and P2Y_6_R [85], P2Y_1_R and P2Y_11_R [86, 87], P2Y_1_R with P2Y_2_R, P2Y_12_R and P2Y_13_R, P2Y_2_R with P2Y_12_R and P2Y_13_R, and P2Y_12_R with P2Y_13_R [83] and P2Y_1_R with P2Y_2_R or P2Y_4_R [88]. Changes in the properties of P2YR following formation of heterodimers have also been reported. Coexpression of P2Y_1_R and P2Y_11_R caused large changes in the potency of the P2Y_1_R antagonist, MRS2179, and the P2Y_11_R antagonist, NF157 [86]. Furthermore, the P2Y_11_R does not normally undergo agonist-induced endocytosis which could now be internalised by ATP. Ala87 of the P2Y_11_R was subsequently revealed to play a crucial role in the interaction with P2Y_1_R [87]. Similarly, the coexpression of P2Y_1_R and P2Y_12_R led to a change in their pharmacological and signalling properties [89].

#### P2YR-adenosine receptor heterodimers

When coexpressed, recombinant P2Y_1_R and P2Y_2_R co-immunopreciptated with adenosine A_1_ receptors [90, 91], P2Y_2_R co-immunopreciptated with A_2A_ receptors [92] and P2Y_1_R, P2Y_2_R, P2Y_12_R and P2Y_13_R generated FRET signals with A_1_ and A_2A_ receptors [83], indicating formation of heterodimers. In the former case, the binding affinities of P2Y_1_R ligands increased and those of A_1_ ligands decreased and coupling to Gq and Gi altered [90]. Ligand binding was unaffected, however, in P2Y_2_R/A_1_ receptor heterodimers, though coupling to Gq and Gi was altered [91]. Native receptors in rat brain also appear to interact in vivo, as A_1_ receptors co-immunopreciptated with P2Y_1_R [93] and P2Y_2_R [94].

#### Other P2YR heterodimers

P2YR also heterodimerise with non-purine receptors, including P2Y_1_R and P2Y_2_R with the M71 olfactory receptor [92], P2Y_12_R with PAR4, but not PAR1 [95, 96] and P2Y_6_R with the angiotensin AT1 receptor [97]. These interactions may have important physiological and pathophysiological roles, as P2Y_1_R and P2Y_2_R appeared to increase the expression of M71, whilst P2Y_6_R promoted angiotensin II-induced hypertension.

### Subtype-selective agonists

Compared to P2XR, more highly subtype-selective P2YR agonists and antagonists have been developed, which is perhaps unsurprising in view of the less conserved nature of the agonist-binding pocket of P2YR. Here, I will focus on those compounds that have been most useful experimentally or therapeutically.

MRS2365 is a highly selective agonist at P2Y_1_R, and MRS2179, MRS2279, MRS2500 and BPTU are potent, selective P2Y_1_R antagonists, whereas clopidogrel, prasugrel, ticlodipine ticagrelor, cangrelor and AZD1283 are all antagonists at P2Y_12_R [41, 42]. In vivo, ADP activates P2Y_1_R and P2Y_12_R in platelets to induce aggregation and thrombus formation, and several of the P2Y_12_R antagonists are widely used as anti-thrombotic agents.

The P2Y_2_R agonist, Diquafasol (UP4U, INS365), stimulates secretion of water and mucin by conjunctival epithelial and goblet cells in the eye and is approved for treatment of dry eye syndrome in South Korea and Japan. AR-C118925XX is the first potent (pA_2_ = 8.43) [98] and highly selective competitive P2Y_2_R antagonist to become commercially available [99], and as such, it is likely to be used widely experimentally. Other potent competitive antagonists that display a degree of subtype selectivity include NF340 at P2Y_11_R (pA_2_ = 8.02) [100], MRS2211 at P2Y_13_R (pA_2_ = 6.3) [101] and PPTN at P2Y_14_R (pK_i_ = 10.1) [102]. MRS2578, in contrast, blocks P2Y_6_R in an irreversible or slowly reversible manner [103]. These antagonists have all been used to investigate the role of P2YR subtypes in a wide range of tissues and cell types.

## Conclusion

It is now over 40 years since Geoff Burnstock proposed that purine nucleotides produce their pharmacological actions through P2R. Since then, this single poorly defined type with few selective ligands and no antagonists has become a large family of multiple subtypes, each with clear and definable properties. We now have detailed knowledge of the tertiary and quaternary structures of individual P2X and P2Y subtypes and highly selective agonists and antagonists that can be used to determine their physiological and pathophysiological roles. P2YR ligands are in clinical use, and numerous other potential therapeutic actions are under study. In addition, cellular release of ATP can now be followed in real time [104], and a family of ecto-enzymes that metabolise extracellular purines and pyrimidine nucleotides has been extensively characterised [105]. This would not have happened without Geoff Burnstock.
